# Natural Enemies Delay Insect Resistance to Bt Crops

**DOI:** 10.1371/journal.pone.0090366

**Published:** 2014-03-03

**Authors:** Xiaoxia Liu, Mao Chen, Hilda L. Collins, David W. Onstad, Richard T. Roush, Qingwen Zhang, Elizabeth D. Earle, Anthony M. Shelton

**Affiliations:** 1 Department of Entomology, China Agricultural University, Beijing, China; 2 Department of Entomology, Cornell University, New York State Agricultural Experiment Station, Geneva, New York, United States of America; 3 Department of Entomology, University of Illinois, Urbana, Illinois, United States of America; 4 Melbourne School of Land and Environment, University of Melbourne, Victoria, Australia; 5 Department of Plant Breeding and Genetics, Cornell University, Ithaca, New York, United States of America; French National Institute for Agricultural Research (INRA), FRANCE

## Abstract

We investigated whether development of resistance to a *Bt* crop in the presence of a natural enemy would be slower than without the natural enemy and whether biological control, in conjunction with a *Bt* crop, could effectively suppress the pest population. Additionally, we investigated whether insecticide-sprayed refuges of non-*Bt* crops would delay or accelerate resistance to the *Bt* crop. We used a system of *Bt* broccoli expressing Cry1Ac, a population of the pest *Plutella xylostella* with a low frequency of individuals resistant to Cry1Ac and the insecticide spinosad, and a natural enemy, *Coleomegilla maculata*, to conduct experiments over multiple generations. The results demonstrated that after 6 generations *P. xylostella* populations were very low in the treatment containing *C. maculata* and unsprayed non-*Bt* refuge plants. Furthermore, resistance to *Bt* plants evolved significantly slower in this treatment. In contrast, *Bt* plants with no refuge were completely defoliated in treatments without *C. maculata* after 4–5 generations. In the treatment containing sprayed non-*Bt* refuge plants and *C. maculata*, the *P. xylostella* population was low, although the speed of resistance selection to Cry1Ac was significantly increased. These data demonstrate that natural enemies can delay resistance to *Bt* plants and have significant implications for integrated pest management (IPM) with *Bt* crops.

## Introduction

The commercialization of plants expressing insecticidal crystal (Cry) proteins from *Bacillus thuringiensis* (*Bt*) for insect management has revolutionized agriculture and become a major tool for integrated pest management (IPM) programs [1]–[2]. In 2011, *Bt* crops were grown on nearly 70 million ha in 27 countries in 2012 [3]. *Bt* crops have provided economic benefits to growers and reduced the use of other insecticides [1], [4]–[6], suppressed pest populations on a regional basis [7]–[9], conserved natural enemies [10] and promoted biological control services in agricultural landscapes [6]. However, the development of insect resistance is a major threat to the sustainable use of *Bt* crops [11]–[12].

Since *Bt* crops were first commercialized in 1996, there is evidence that three lepidopteran pests have evolved resistance to *Bt* crops in the open field [13]–[15] and one case of a coleopteran pest [16]. Resistance to *Bt* plants is a serious concern, but the relatively few number of cases is in stark contrast to many cases of resistance to conventional insecticides, which has occurred much more rapidly [17]. Commonly proposed reasons for the few confirmed cases of resistance to *Bt* plants are the high dose of *Bt* proteins expressed in plants and the use of refuges of non-*Bt* plants that can serve as a pool of *Bt* susceptible alleles in the population [18]–[19].

Another possible reason for the relatively few cases of resistance to *Bt* plants could be their safety to natural enemies that help suppress pest populations. Numerous studies have investigated the effects of *Bt* crops and Cry proteins on natural enemies (predators and parasitoids) in the laboratory and field [20]–[21]. A meta-analysis has confirmed the safety of *Bt* proteins, especially when compared to traditional insecticides [10]. When negative effects on natural enemies have been observed with *Bt* proteins, they appear to be due to the poor quality of the host and not the Cry protein [22], but see Desneux et al., 2010 [23]. The safety of several *Bt* proteins has been verified in tritrophic studies conducted with *Bt*-resistant or non-susceptible herbivores that avoided the problems of prey-quality in some previous studies [22]. Allowing *Bt*-resistant hosts to ingest *Bt* proteins and then feeding the hosts to natural enemies (both predators and parasitoids) has revealed no effects on the natural enemies [24]–[26]. However, some reports continue to suggest natural enemies may be harmed by *Bt* proteins [27], but these have been challenged [28].

The conservation of natural enemies by the use of *Bt* plants could also influence the development of resistance to *Bt* crops. This question was first studied by Gould et al. [29] in their conceptual and mathematical models on tritrophic interactions of a plant, an herbivore and a natural enemy. Their simplest conclusion was that natural enemies that increase differential fitness between susceptible and resistant phenotypes on host plants will accelerate resistance; those that decrease the differential will delay resistance. Johnson et al. [30]–[31] carried out controlled studies of a parasitoid and a pathogenic fungus that attack *Heliothis virescens* on *Bt* tobacco and concluded that the parasitoid would likely delay the development of resistance to transgenic tobacco, while the pathogen would likely promote the development of resistance. Mallampalli et al. [32] discovered that different prey species of a generalist predator had different effects on the development of resistance by *Leptinotarsa decemlineata* to *Bt* potato: the presence of one prey species delayed resistance while the other accelerated resistance. Heimpel et al. [33] reported that the form of egg mortality could influence the rate of resistance, but the importance of egg mortality depended on other ecological processes in the pest population. Other simulation models reported that natural enemies could slow insect resistance to *Bt* crops or *Bt* pesticides [34]–[36]. As this summary indicates, the literature contains suggestions that natural enemies could delay or accelerate resistance, depending on whether there is a differential impact on susceptible or resistant phenotypes.

In the present study we used a unique system, composed of broccoli plants transformed to express Cry1Ac protein, a population of *Plutella xylostella*, a global pest of crucifers [37] with a low frequency of resistant individuals to Cry1Ac and the insecticide spinosad, and the predaceous ladybird beetle, *Coleomegilla maculata*, to conduct a multigenerational study in the greenhouse. Our objectives were to determine: (1) if a natural enemy can delay the development of insect resistance to a *Bt* crop; and (2) if biological control in conjunction with *Bt* crops can effectively suppress the pest population. In addition, to simulate field-realistic conditions for both the predator and prey, we sprayed refuges with insecticide in some treatments, but not others, and observed those effects on the development of insect resistance.

## Results

### Population Density of *P. xylostella*

**Figure 1:**
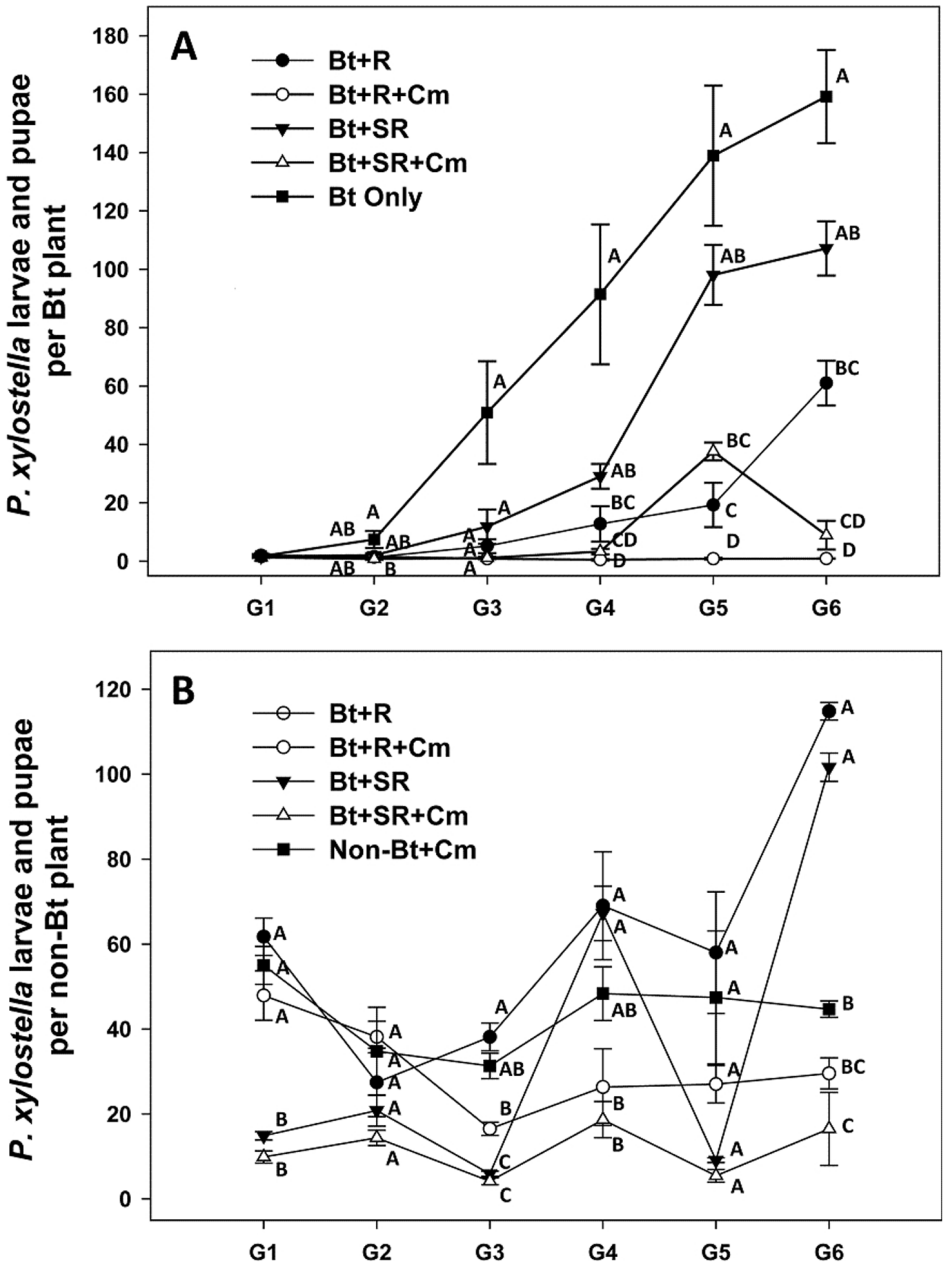
*Plutella xylostella* populations on *Bt* plants (A) and on non-*Bt* refuge plants (B) in greenhouse cages (Means ± SEM). Bt+R: 75% *Bt* and 25% non-*Bt* refuge plants; Bt+R+Cm: 75% *Bt* and 25% non-*Bt* refuge plants with *C. maculata*; Bt+SR: 75% *Bt* and 25% spinosad-sprayed non-*Bt* refuge plants; Bt+SR+Cm: 75% *Bt* and 25% spinosad-sprayed non-*Bt* refuge plants with *C. maculata*; Bt Only: 100% *Bt* plants only, Non-Bt+Cm: 100% non-*Bt* refuge plants with *C. maculata*. Different letters within the same generations denote significant differences (*P*<0.05, one-way ANOVA, Games-Howell test for G3 and G6 in A, G1, G3 and G5 in B, other comparsions by Tukey HSD test).

The predator, presence of a refuge, and use of a spray on the refuge each influenced the population dynamics of *P. xylostella* per *Bt* plant over the 6 generations of the experiment. A repeated-measures ANOVA, with generation and treatment as factors, yielded a significant effect for generations (*F*_3.164_ = 77.101, *P*<0.001), for treatments (*F*_4_ = 31.788, *P*<0.001), and the interaction term generation*treatments (*F*_12.656_ = 16.250, *P*<0.001). During the 1^st^ generation, few *P. xylostella* were found on *Bt* plants (Fig. 1A), and there were no significant differences between treatments using one-way ANOVA (*F*_4_ = 0.258, *P* = 0.900). By the 2^nd^ generation, there was an average of 7 *P. xylostella* larvae and pupae per *Bt* plant in the treatment with only *Bt* plants, but the number of *P. xylostella* was still about 1 per plant in the other treatments (*F*_4_ = 3.767, *P* = 0.026). *Bt* plants were completely defoliated and control failure was evident in the *Bt* plant-only treatment at the 3^rd^ generation when the number of *P. xylostella* had increased to 51 per *Bt* plant (*F*_4_ = 8.667, *P* = 0.001).

At the 4^th^ generation, the number of *P. xylostella* increased to 12 per *Bt* plant in treatment Bt+R (75% *Bt* plants +25% non-*Bt* refuge plants) and 29 per *Bt* plant in Bt+SR (75% *Bt* plants +25% spinosad-sprayed non-*Bt* refuge plants), significantly higher than those in treatments Bt+R+Cm (75% *Bt* plants +25% non-*Bt* refuge plants + predator) and Bt+SR+Cm (75% *Bt* plants +25% spinosad- sprayed non-*Bt* refuge plants + predator) (*F*_4_ = 21.294, *P*<0.001). There was still only about 1 *P. xylostella* per *Bt* plant in treatment Bt+R+Cm at the 5^th^ and 6^th^ generations, significantly lower than other treatments (5^th^ generation: *F*_4_ = 59.203, *P*<0.001; 6^th^ generation: *F*_4_ = 61.164, *P*<0.001). Most importantly, over the 6 generations of the test, only treatment Bt+R+Cm maintained <2 *P. xylostella* per *Bt* plant at each generation, suggesting the important role that the predator played in maintaining a low pest population.

When spinosad was used (treatment Bt+SR+Cm), the pest population was also maintained at a low level, except for a flare-up in the 5^th^ generation when the *P. xylostella* population was >40 per *Bt* plant. Without the use of the predator (Bt+R), the pest population gradually rose and peaked at 61 per plant by the 6^th^ generation, compared to being maintained at about 1 for all generations when the predator was used (Bt+R+Cm). When the predator was replaced by the insecticide spinosad (Bt+SR), the pest population increased more rapidly and peaked at 107 per plant in the 6^th^ generation, again showing the strong and lasting benefit of the predator in maintaining a low pest population.

Examining the pest population on the refuge plants in the cages provided another indication of the overall performance of the treatments. Using repeated measures analysis, there were significant differences for generations (*F*_3.740_ = 29.341, *P*<0.001), for treatments (*F*_4_ = 60.288, *P*<0.001), and the interaction term generation*treatments (*F*_14.960_ = 10.071, *P*<0.001). On refuge plants, the density of *P. xylostella* varied between 5–120 *P. xylostella* per plant between generations and treatments (Fig 1B). Only the populations in Bt+SR+Cm, which combined *Bt* plants, spinosad and the predator, were consistently the lowest and did not exceed 20 per plant in any generation. Use of the predator alone (Bt+R+Cm) maintained a relatively low and stable pest population over all 6 generations. In treatments of 100% non-*Bt* refuge plants, *P. xylostella* densities exceeded 100 per plant at each generation despite keeping only 3 defoliated plants with their larvae and pupae in each cage to reduce the overall population. Therefore, we did not include the treatment of only non-*Bt* plants in Figure 1B.

### Population Density of *C. maculata*

**Figure 2:**
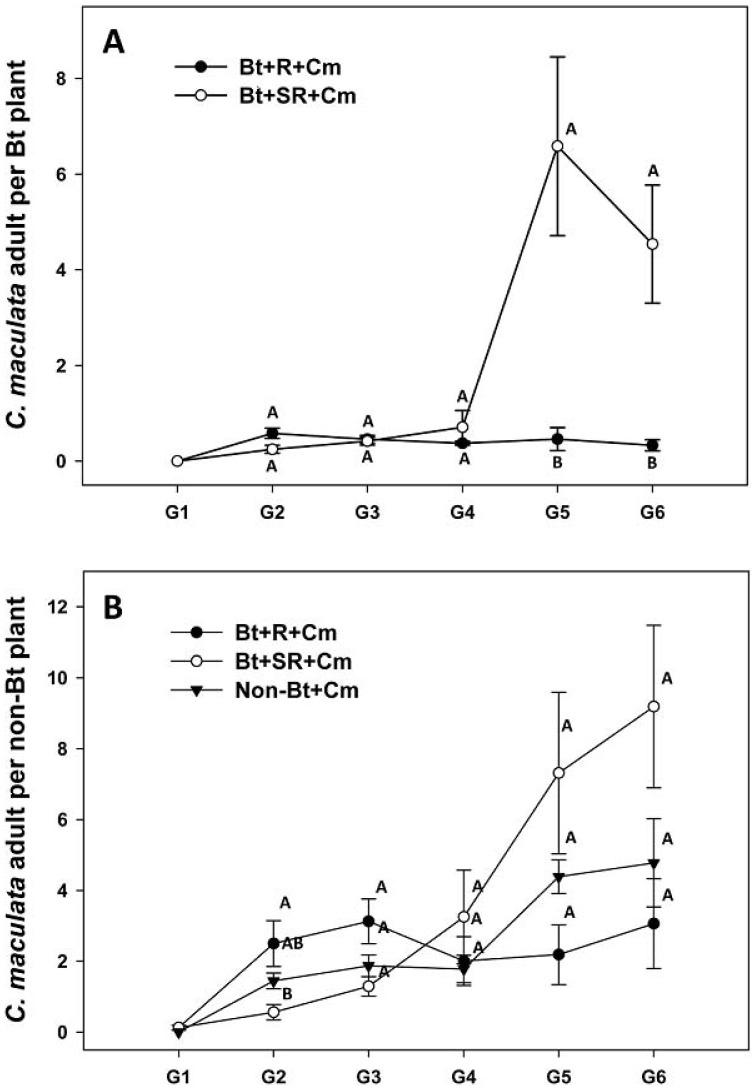
*Coleomegilla maculata* population on *Bt* plants (A) and on non-*Bt* refuge plants (B) in greenhouse cages (Means ± SEM). Bt+R+Cm: 75% *Bt* and 25% non-*Bt* refuge plants with *C. maculata*; Bt+SR+Cm: 75% *Bt* and 25% spinosad-sprayed non-*Bt* refuge plants with *C. maculata*; Non-Bt+Cm: 100% non-*Bt* refuge plants with *C. maculata*. Different letters within the same generations denote significant differences (*P*<0.05, one-way ANOVA, Tukey HSD test).

In treatments Bt+R+Cm and Bt+SR+Cm, predator populations generally increased as the pest, *P. xylostella*, populations increased. A repeated-measures ANOVA yielded a significant effect for generations (*F*_1.838_ = 11.873, *P* = 0.002), for treatments (*F*_1_ = 9.410, *P* = 0.022), and the interaction term generation*treatments (*F*_1.838_ = 11.225, *P* = 0.003). Only a few *C. maculata* adults were found in the 1^st^ generation because only 3 pairs were released at the start of experiment (Fig. 2A). Predator populations on *Bt* plants in treatment Bt+R+Cm remained about 1 per plant because of the low pest population, especially on *Bt* plants through the 6^th^ generation (Fig. 1A). In treatment Bt+SR+Cm, predator populations remained about 1 per plant until the 5^th^ generation when they increased to >4.5 in the 5^th^ and 6^th^ generations (Fig. 2A). Mean values for the 5th and 6th generations by the independent-test between the two treatments, respectively, differed significantly (G5: *t*_(6)_ = 4.562, *P* = 0.004; G6: *t*_(6)_ = 6.268, *P* = 0.001). This likely maintained the pest population on the *Bt* plants at a low population (Fig. 1A), although resistance increased (Table 1).

**Table 1:** Survival on Cry1Ac leaf (%) (Means ± SEM) of *Plutella xylostella* larvae from adults taken from cages.

Treatments	Generation			
	G3	G4	G5	G6
Bt+R	3.3±1.86 A	19.0±6.61 B	32.5±14.55 BC	39.5±14.06 B
Bt+R+Cm	4.7±3.18 A	7.0±3.06 AB	5.0±3.51 AB	6.0±2.86 A
Bt+SR	29.3±4.01 B	56.0±4.74 C	87.5±1.44 D	83.8±3.07 D
Bt+SR+Cm	3.3±1.93 A	20.7±2.19 B	36.8±6.85 C	72.8±3.28 D
Bt Only	74.9±5.46 C	72.0±6.76 C	90.5±3.97 D	93.5±1.19 D
Non-Bt Only	1.0±0.58 A	1.3±0.33 A	1.0±0.58 A	3.7±1.33 A
Non-Bt+Cm	0.7±0.33 A	1.0±0.58 A	4.7±1.86 AB	2.3±1.86 A

Bt+R: 75% *Bt* and 25% non-*Bt* refuge plants; Bt+R+Cm: 75% *Bt* and 25% non-*Bt* refuge plants with *C. maculata*; Bt+SR: 75% *Bt* and 25% spinosad-sprayed non-*Bt* refuge plants; Bt+SR+Cm: 75% *Bt* and 25% spinosad-sprayed non-*Bt* refuge plants with *C. maculata*; Bt Only: only *Bt* plants; Non-Bt Only: only non-*Bt* plants; Non-Bt+Cm: only non-*Bt* plants with *C. maculata*.

Different letters within the same column denote significant differences (*P*<0.05, one-way ANOVA, Tukey's test).

Predator populations were generally higher on the refuge plants (Fig. 2B) than on the *Bt* plants (Fig. 2A), likely reflecting the higher pest density on these plants. For the three treatments Bt+R+Cm, Bt+SR+Cm, and non-Bt+Cm, there were no significant differences in predator density in most generations. The repeated measures showed significant effects for generations (*F*_3.460_ = 12.234, *P*<0.001) and the interaction term generation*treatments (*F*_6.920_ = 3.241, *P* = 0.012), but not for the treatments (*F*_2_ = 2.181, *P* = 0.175).

### Resistance to Cry1Ac

Survival of *P. xylostella* larvae indicates resistance and, on *Bt* broccoli, it was significantly affected when analyzed using repeated-measures for generations (*F*_2.714_ = 35.792, *P*<0.001), for treatments (*F*_6_ = 81.199, *P*<0.001), and the interaction term generation*treatments (*F*_16.282_ = 6.719, *P*<0.001). Resistance levels reached 74.9% after only 3 generations in the treatment with *Bt* plants only, significantly greater than the survival in other treatments (*F*_6_ = 50.049, *P*<0.001) and >2x the next highest treatment of Bt+SR (75% *Bt* plants + 25% spinosad-sprayed non-*Bt* refuge plants) (Table 1). The high rate of resistance in the treatment with only *Bt* plants was sustained through the 6^th^ generation. In treatment Bt+SR, resistance also developed rapidly, with 56.0% *P. xylostella* surviving at the 4^th^ generation, significantly greater than the treatments Bt+R, Bt+R+Cm, Bt+SR+Cm and the control cages (Non-Bt Only and Non-Bt+Cm) (*F*_6_ = 33.319, *P*<0.001). The survival rates were 4–7% in treatment Bt+R+Cm (with predators) at all generations, but increased to 32.5% and 39.5% in treatment Bt+R (without predators) at the 5^th^ and 6^th^ generations, respectively. There were significant differences among treatments (5^th^ generation: *F*_6_ = 26.302, *P*<0.001; 6^th^ generation: *F*_6_ = 38.850, *P*<0.001) with <10% survival in Bt+R+Cm, Non-Bt Only and Non-Bt+Cm, and >70% survival in Bt+SR, Bt+SR+Cm and Bt Only, while Bt+R had nearly 40% survival in the 6^th^ generation. These significant differences at the 6^th^ generation among Bt+R, Bt+R+Cm and Bt+SR+Cm highlight the ability of the predator to slow the rate of resistance to *Bt* plants. Treatment Bt+R+Cm, which included the predator, had the lowest survival (6.0%) while the treatment without the predator (Bt+R) had 39.5% survival. Including the predator but using spinosad (Bt+SR+Cm), which has some toxicity to Cm [38], increased the development of resistance to *Bt* plants as indicated by the 72.8% survival (Table 1).

### Resistance to Spinosad

In the treatments in which spinosad was used (Bt+SR and Bt+SR+Cm), only low levels of survival of *P. xylostella* were detected. Survival of individuals removed from cages and fed spinosad-treated leaves was only about 1% at the 4^th^ and 6^th^ generations.

## Discussion

A large body of literature has evaluated the potential ecological risks of *Bt* crops to non-target organisms including natural enemies of insect pests [1], [2], [22], [39]. However, as is the case with conventional insecticides, the interaction of natural enemies and resistance development in their hosts has received far less attention.

Our present results indicate that the predator, *C. maculata*, combined with non-*Bt* and unsprayed refuge plants, delayed resistance in the *P. xylostella* population to *Bt* broccoli plants, when compared to treatments without the predator. Some theoretical work predicted a similar conclusion, that natural enemies could slow the development of insect resistance to *Bt* crops or *Bt* sprayed on crops [30], [34]–[36]. The present work, conducted over multiple generations, adds empirical evidence to the theory that natural enemies can slow the development of resistance to *Bt* crops.

Arpaia et al. [35] reported that the distribution of *C. maculata* in potato fields is driven by the prey's density but, in spite of this aggregation, a significant number of them occur in areas where food density is low. In the present study, we observed some *C. maculata* adults and larvae on *Bt* plants, although the prey density was very low on these plants. Still the likelihood of a *Bt*-resistant *P. xylostella* individual reaching adulthood on transgenic plants will be lowered if there are natural enemies on *Bt* plants, and this, in turn, will slow the rate at which the resistance alleles increase in frequency. In our tests, *C. maculata* were found on *Bt* plants (Fig. 2B) where they likely encountered *Bt*-resistant *P. xylostella* and removed them, thus eliminating resistance alleles from the population. This is the likely cause of the slower rate of resistance in the treatment with the refuge and predator. Although spinosad is considered relatively non-toxic to natural enemies, it does have a toxic effect on *C. maculata*, although much less than “harder insecticides” [38].

Widespread planting of *Bt* crops places strong selection pressure on pest populations, which could result in resistance to *Bt* and control failure [11], [17]–[19]. Currently, an accepted cornerstone of resistance management programs for *Bt* crops in the United States, Australia, and elsewhere, is a refuge consisting of plants free of *Bt* insecticidal proteins, thus allowing *Bt*-susceptible alleles to persist in the population. When resistance is recessive, as has been the case so far for many key pests, then matings between resistant individuals emerging from *Bt* plants and those from refuge plants will greatly reduce the possibility of creating homozygous resistant offspring that can damage *Bt* plants [17]. In the United States, the Environmental Protection Agency (EPA) mandates non-*Bt* refuges of different sizes for different *Bt* crops.

Empirical data have demonstrated the usefulness of refuges for *Bt* crops. Tang et al. [40] reported that pest population growth was influenced by refuge size, with the highest populations occurring in treatments that had either no refuge plants or all refuge plants. That study also confirmed other models [41] that the development of resistance was inversely proportional to the size of the refuge. Most importantly, Tang et al. [40] demonstrated that a balance could be struck by refuge sizes that would delay resistance while at the same time limit the growth of the pest population and damage to the crop, as has been observed in the field [7], [9].

In the present study, results indicate that refuges slow resistance, but a sprayed refuge could accelerate it. These findings are in line with our previous field study [42]. In the treatment with only *Bt* plants without refuge plants, control failure and high insect densities were observed in the 3^rd^ generation (two replications) and the 4^th^ generation (the other two replications). Plants in the treatment of spinosad-sprayed non-*Bt* broccoli plants (Bt+SR) were completely defoliated between the 4^th^ and 5^th^ generations.

In the treatment (Bt+SR+Cm) in which the predator and spinosad were used, 36.8% of the pest population survived on *Bt* plants by the 5^th^ generation and by the 6^th^ generation that had increased to 72.8% (Table 1). Spinosad killed most susceptible *P. xylostella* on refuge plants, and not enough susceptible individuals mated with the resistant individuals on *Bt* plants. Therefore, resistance development was quick although there were *C. maculata* in the treatment (Bt+SR+Cm). However, the pest populations were very low in this treatment with 16.5 *P. xylostella* larvae per refuge plant (Fig. 1A) and 8.9 per *Bt* plant (Fig. 1B) at the 6^th^ generation. In the 6^th^ generation, we found >100 *C. maculata* in each cage. This indicates that the high predator population controlled *P. xylostella* density to a low level, despite the fact that the pest had developed resistance to the *Bt* toxin.

While farmers are concerned with reducing the likelihood of resistance, they are more immediately concerned with lowering the pest population to avoid crop injury. It is well worth noting that in our experiments we not only saw the lowest rate of resistance development in the prey when the predator was not decimated by the use of an insecticide, but also the lowest and least fluctuating pest population on *Bt* plants (Fig. 1A) and low and stable pest populations on non-*Bt* plants in the refuge (Fig. 1B). Thus, our data suggest that farmers can have sustainable management of pests if they combine *Bt* plants with biological control.

In conclusion, this study provides empirical evidence to confirm the theory that natural enemies can delay resistance development to *Bt* plants, but also demonstrates that it can do so while maintaining a low pest density and low crop damage. Non-*Bt* refuges are necessary to delay resistance to *Bt* plants, but spraying refuges could accelerate resistance if sprays reduce the function of important biological control agents of the pest. We suggest that host-plant resistance with *Bt* plants and biological control can be fully compatible within an overall integrated pest management (IPM) program. Our results have significant implications for IPM and insect resistance management (IRM) for *Bt* crops.

## Methods

### Insects

Three strains of *Plutella xylostella* were used to create a hybrid population for the cage tests: the susceptible Geneva 88 (SS), the Cry1Ac-resistant (Cry1Ac-RR), and the spinosad-resistant (Pearl-RR) [24], [40], [43] strains. The hybrid population was created by releasing 100 F1 RS1 (G88 female × Cry1Ac-RR male), 100 RS2 (G88 female × Pearl-RR male) and 300 G88 moths into a cage. The total number of moths in the cage was 500 with a 1∶1 ratio for female and male moths from each strain. Eggs were collected from the cage and put on artificial diet to rear F1 larvae. About 1,000 moths from F1-F4 were used to produce a synthetic population. F5 pupae were used in the selection experiments. The expected allele frequency of the synthetic population (square root of survival rate) was 0.1 for Cry1Ac and spinosad resistance. The mean survival of F5 larvae was 0.033 on Cry1Ac plants and 0.025 on spinosad-sprayed plants. Therefore, the actual initial allele frequency at the start of the experiment was estimated to be 0.057 (square root of 0.033) for Cry1Ac resistance and 0.050 (square root of 0.025) for spinosad resistance [43]. While this is higher than would be expected in the field initially when *Bt* crops are released (perhaps 10^−3^ or lower [41]), we expected that these initial frequencies would allow us to see differences among the treatments in a reasonable time frame.

Larvae and adult *C. maculata* were obtained from DuPont Pioneer (Johnston, IA) and maintained in a climatic chamber at 27±1°C, 65±5% RH and 16∶8 L: D at Cornell University's Department of Entomology at Geneva, NY. Both larvae and adults were reared on decapsulated eggs of brine shrimp, *Artemia franciscana*, (Brine Shrimp Direct, Ogden UT) and 1.5% agar solution provided separately as a water source.

### Transgenic broccoli plants and insecticide

We used *Brassica oleracea* producing high levels of Cry1Ac for our *Bt* broccoli plants [44]. *Bt* broccoli plants with 8 true leaves were used, and analysis by ELISA indicated that the Cry1Ac protein level was 12.33±1.62 µg/g fresh leaf tissue (n = 7). To ensure the activity of the *Bt* broccoli, the plants were screened with *P. xylostella* neonates of F1 heterozygotes (G88 × Cry1Ac-RR) when plants were 4 to 5 wk old [43]. The Cry1Ac plants that killed 100% of neonates of F1 heterozygotes (SS x Cry1Ac-RR), indicating high levels of expression in the *Bt* plants, were used in the greenhouse experiments. Non-*Bt* broccoli (cv. Packman) was used in the refuge.

A commercial formulation of spinosad (SpinTor 2 SC, 240 g [AI]/Liter) was used. Refuge plants in the treatments B1 and B2 were sprayed in the cages using a small hand-held sprayer. The Cry1Ac plants were covered during the sprays to avoid drift. The concentration we used was 90 ppm, which was the lowest field dose listed on the insecticide label. The insecticide-treated refuges were sprayed at the 1^st^, 3^rd^ and 5^th^ generations in order to keep the *P. xylostella* population in check in the cages.

### Experimental designs

The selection experiment was conducted in greenhouses at Cornell University's New York State Agricultural Experiment Station and followed the general procedures used in previous studies [40], [43]. Each cage was 1.8 m long×0.9 m wide×1.7 m high and constructed of nylon netting. Adults, but not larvae, could easily move between the different broccoli types, which were separated by a nylon-netting barrier (0.9 m high). This arrangement simulated adjoining fields with frequent inter-field movement by adults but negligible movement of larvae.

Seven treatments were included in the experiment (Table 2). There were 16 plants total in each cage (12 *Bt* plants plus 4 non-*Bt* refuge plants). 250 F4 pupae of the synthetic *P. xylostella* population were released into each cage. Three pairs of 1-week old *C. maculata* were released into cages of the treatments Bt+R+Cm, Bt+SR+Cm and non-Bt+Cm when the *P. xylostella* larvae were 2^nd^ instars. During the pupal period, all old plants were cut at the base of the stem, and plants and pots (which might have pupae on them) were kept in the cages for a week in order to allow adults to emerge. New plants were introduced into the cage to provide foliage for egg laying or existing larvae that had defoliated plants. There were four replications (cages) of *Bt*-plant treatments (Bt+R, Bt+R+Cm, Bt+SR, Bt+SR+Cm and Bt Only) and three of the controls (Non-Bt Only and non-Bt+Cm).

**Table 2:** Experimental treatments.

Group	Treatments	Replications
Bt+R	75% Bt plants and 25% non-Bt refuge plants	4
Bt+R+Cm	75% Bt plants and 25% non-Bt refuge plants and *C. maculata*	4
Bt+SR	75% Bt plants and 25% non-Bt refuge plants treated with spinosad	4
Bt+SR+Cm	75% Bt plants and 25% non-Bt refuge plants treated with spinosad and *C. maculata*	4
Bt Only	100% Bt plants only	4
Non-Bt Only	100% Non-Bt refuge plants	3
Non-Bt+Cm	100% Non-Bt refuge plants and *C. maculata*	3

R: refuge.

SR: refuge with spinosad.

Cm: *C. maculata*.

### Data collection

Older larvae (primarily 3rd or 4th instars) and pupae of *P. xylostella*, and pupae and adults of *C. maculata* on broccoli plants were counted every generation when larval and pupal densities peaked. To test for resistance, 30–40 larvae from non-*Bt* refuge plants were collected from each cage at the 3^rd^, 4^th^ and 5^th^ generations. The larvae were reared on diet in the laboratory, and then adults were allowed to mate and eggs were harvested and tested as described below. At the 6^th^ generation, we collected at least 60 pupae on refuge plants from most of the cages. The survival of 2^nd^ instars derived from the pupae collected in each cage was tested on Cry1Ac broccoli leaf disks in 30-ml plastic cups [43]. For each cage, a total of 100 larvae in 10 replications were tested on *Bt* broccoli, and non-*Bt* broccoli was used as a control. We also tested survival of 2^nd^ instars derived from B1 and B2 treatments on spinosad-dipped broccoli leaf disks in 30-ml plastic cups at the diagnostic dose of 10 ppm [45]. A total of 100 larvae were tested (10 replications, 10 larvae/rep) for each cage. For both Cry1Ac plants and spinosad treatments, survival was determined after 3 days at 27±1°C, 50±10% RH and 16∶8 L:D photoperiod.

### Statistical analysis

All statistical analyses were conducted using SPSS 17.0 Windows [46]. Descriptive statistics are given as mean values and standard errors of the mean. Because the data fit the assumptions for parametric analysis, the repeated measures ANOVA was used with the factors of generations and treatments for analysis of the population of *P. xylostella* on *Bt* and non-*Bt* plants and the survival on Cry1Ac leaves of *P. xylostella* larvae from adults taken from cages. The survival rates were transformed by square root before analysis. For each generation, the differences of resistance development and the population of *P. xylostella* and *C. maculata* were analyzed by one-way ANOVA, and means were compared by Tukey HSD, if the data fit homoscedasticity, and Games-Howell test if not. Differences of the mean values in the population of *C. maculata* per *Bt* plants between the treatments of refuge + *C. maculata* and sprayed-refuge + *C. maculata* were examined by independent t-tests. In all tests *P* values <0.05 were considered significant.

## References

[1] Shelton AM, Zhao J-Z, Roush RT (2002) Economic, ecological, food safety, and social consequences of the deployment of Bt transgenic Plants. Annu Rev Entomol 47: 845–881. 11729093 10.1146/annurev.ento.47.091201.145309

[2] Romeis J, Shelton AM, Kennedy GG (2008) Integration of insect-resistant genetically modified crops within IPM programs. Dordrecht, Springer. 441 pp.

[3] James C (2012) Global status of commercialized transgenic crops: Bt cotton, ISAAA Briefs No. 44. Ithaca, NY: International Service for the Acquisition of Agri-biotech Applications.

[4] Qaim M, Pray CE, Zilberman D (2008) Economic and social considerations in the adoption of Bt crops. In: Romeis J, Shelton AM, Kennedy GG, editors. Integration of insect-resistant genetically modified crops within IPM programs. Dordrecht: Springer. pp 329–356.

[5] Kathage J, Qaim M (2012) Economic impacts and impact dynamics of Bt cotton in India. Proc Natl Acad Sci USA 109: 11652–11656. 22753493 10.1073/pnas.1203647109 PMC3406847

[6] Lu Y, Wu K, Jiang Y, Guo Y, Desneux N (2012) Widespread adoption of Bt cotton and insecticide decrease promotes biocontrol services. Nature 487: 362–365. 22722864 10.1038/nature11153

[7] Carrière Y, Ellers-Kirk C, Sisterson M, Antilla L, Whitlow M, et al (2003) Long-term regional suppression of pink bollworm by *Bacillus thuringiensis* cotton. Proc Natl Acad Sci USA 100: 1519–1523. 12571355 10.1073/pnas.0436708100 PMC149864

[8] Wu KM, Lu YH, Feng HQ, Jiang YY, Zhao J-Z (2008) Suppression of cotton bollworm in multiple crops in China in areas with Bt toxin-containing cotton. Science 321: 1676–1678. 18801998 10.1126/science.1160550

[9] Hutchison WD, Burkness EC, Mitchell PD, Moon RD, Leslie TW, et al (2010) Areawide suppression of European corn borer with Bt maize reaps savings to non-Bt maize growers. Science 330: 222–225. 20929774 10.1126/science.1190242

[10] Naranjo SE (2009) Impacts of Bt crops on non-target organisms and insecticide use patterns. CAB Reviews: Perspectives in agriculture, veterinary science, nutrition and natural resources 4 : No.011. DOI: 10.1079/PAVSNNR20094011.

[11] Gould F (1998) Sustainability of transgenic insecticidal cultivars: integrating pest genetics and ecology. Annu Rev Entomol 43: 701–726. 15012402 10.1146/annurev.ento.43.1.701

[12] Onstad DW (2008) The future of insect resistance management. In: Onstad DW, editor. Insect resistance management: biology, economics and prediction. London: Academic Press. pp. 289–300.

[13] Van Rensburg JBJ (2007) First report of field resistance by stem borer, *Busseola fusca* (Fuller) to Bt-transgenic maize. S African J Plant Soil 24: 147–151.

[14] Storer NP, Babcock JM, Schlenz M, Meade T, Thompson GD, et al (2010) Discovery and characterization of field resistance to Bt maize: *Spodoptera frugiperda* (Lepidoptera: Noctuidae) in Puerto Rico. J Econ Entomol 103: 1031–1038. 20857709 10.1603/ec10040

[15] Dhurua S, Gujar GT (2011) Field-evolved resistance to Bt toxin Cry1Ac in the pink bollworm, *Pectinophora gossypiella* (Saunders) (Lepidoptera: Gelechiidae) from India. Pest Manag Sci 67: 898–903. 21438121 10.1002/ps.2127

[16] Gassmann AJ, Petzold-Maxwell JL, Keweshan RS, Dunbar MW (2011) Field-evolved resistance to Bt maize by western corn rootworm. PLoS ONE 6(7): e22629. 21829470 10.1371/journal.pone.0022629 PMC3146474

[17] Bates SL, Zhao JZ, Roush RT, Shelton AM (2005) Insect resistance management in GM crops: past, present and future. Nat Biotechnol 23: 57–62. 15637622 10.1038/nbt1056

[18] Tabashnik BE, Van Rensburg JBJ, Carrière Y (2009) Field-evolved insect resistance to Bt crops: definition, theory, and data. J Econ Entomol 102: 2011–2025. 20069826 10.1603/029.102.0601

[19] Huang F, Andow D, Bushman LL (2012) Success of the high-dose/refuge resistance management strategy after 15 years of Bt crop use in North America. Entomol Exp Appl 140: 1–16.

[20] Wolfenbarger LL, Naranjo SE, Lundgren JG, Bitzer RJ, Watrud LS (2008) Bt crops effects on functional guilds of non-target arthropods: a meta-analysis. PLoS ONE 3(5): e2284 DOI:10.1371/journal.pone.0002118 18461164 10.1371/journal.pone.0002118 PMC2346550

[21] Desneus N, Bernal JS (2010) Genetically modified crops deserve greater ecotoxicological scrutiny. Ecotoxicology 19: 1642–1644. 20882339 10.1007/s10646-010-0550-8

[22] Romeis J, McLean MA, Shelton AM (2013) When bad science makes good headlines: the case of Bt maize and regulatory bans. Nat Biotechnol 37: 386–7. 10.1038/nbt.2578 23657387

[23] Desneus N, Ramirez-Romero R, Bokonon-Ganta AH, Bernal JS (2010) Attraction of the parasitoid *Cotesia marginiventris* to host (*Spodoptera frugiperda*) frass is affected by transgenic maize. Ecotoxicology 19: 1183–1192. 20480228 10.1007/s10646-010-0502-3

[24] Chen M, Zhao JZ, Collins HL, Earle ED, Cao J, et al (2008) A critical assessment of the effects of Bt transgenic plants on parasitoids. PLoS ONE 3(5): e2284 DOI: 10.1371/journal.pone.0002284 18523682 10.1371/journal.pone.0002284 PMC2409141

[25] Liu XX, Chen M, Onstad D, Roush R, Shelton AM (2011) Effect of Bt broccoli and resistant genotype of *Plutella xylostella* (Lepidoptera: Plutellidae) on development and host acceptance of the parasitoid *Diadegma insulare* (Hymenoptera: Ichneumonidae). Transgenic Res 20: 887–897. 21181494 10.1007/s11248-010-9471-9

[26] Tian JC, Collins HL, Romeis J, Naranjo SE, Hellmich RL, et al (2012) Using field-evolved resistance to Cry1F maize in a lepidopteran pest to demonstrate no adverse effects of Cry1F on one of its major predators. Transgenic Res 21: 1303–1310. 22373893 10.1007/s11248-012-9604-4 PMC3505541

[27] Lovei GL, Andow DA, Arpaia S (2009) Transgenic insecticidal crops and natural enemies: a detailed review of laboratory studies. Environ Entomol 38: 293–306. 19389277 10.1603/022.038.0201

[28] Shelton AM, Naranjo S, Romeis J, Hellmich RH, Wolt J, et al (2009) Setting the record straight: a rebuttal to an erroneous analysis on transgenic insecticidal crops and natural enemies. Transgenic Res 18: 317–322. 19357987 10.1007/s11248-009-9260-5

[29] Gould F, Kennedy GG, Johnson MT (1991) Effects of natural enemies on the rate of herbivore adaptation to resistant host plants. Entomol Exp Appl 58: 1–14.

[30] Johnson MT, Gould F, Kennedy GG (1997) Effects of natural enemies on relative fitness of *Heliothis virescens* genotypes adapted and not adapted to resistant host plants. Entomol Exp Appl 82: 219–230.

[31] Johnson MT, Gould F, Kennedy GG (1997) Effect of an entomopathogen on adaptation of *Heliothis virescens* populations to transgenic host plants. Entomol Exp Appl 83: 121–135.

[32] Mallampalli N, Gould F, Barbosa P (2005) Predation of Colorado potato beetle eggs by a polyphagous ladybeetle in the presence of alternate prey: potential impact on resistance evolution. Entomol Exp Appl 114: 47–54.

[33] Heimpel GE, Neuhauser C, Andow DA (2005) Natural enemies and the evolution of resistance to transgenic insecticidal crops by pest insects: the role of egg mortality. Environ Entomol 34: 512–526.

[34] Chilcutt CF, Tabashnik BE (1999) Simulation of integration of *Bacillus thuringiensis* and the parasitoid *Cotesia plutellae* (Hymenoptera: Braconidae) for control of susceptible and resistant diamondback moth (Lepidoptera: Plutellidae). Environ Entomol 28: 505–512.

[35] Arpaia S, Gould F, Kennedy G (1997) Potential impact of *Coleomegilla maculata* predation on adaptation of *Leptinotarsa decemlineata* to Bt-transgenic potatoes. Entomol Exp Appl 82: 91–100.

[36] Gassmann AJ, Stock SP, Carrière Y, Tabashnik BE (2006) Effect of entomopathogenic nematodes on the fitness cost of resistance to *Bt* toxin *Cry1Ac* in pink bollworm (Lepidoptera: Gelechiidae). J Econ Entomol 99: 920–926. 16813331 10.1603/0022-0493-99.3.920

[37] Talekar NS, Shelton AM (1993) Biology, ecology and management of the diamondback moth. Annu Rev Entomol 38: 275–301. 10.1146/annurev-ento-010715-023622 26667272

[38] Liu XX, Chen M, Collins HL, Onstad D, Roush R, et al (2012) Effect of insecticides and *Plutella xylostella* (Lepidoptera: Plutellidae) genotype on a predator and parasitoid and implications for the evolution of insecticide resistance. J Econ Entomol 105: 354–362. 22606803 10.1603/ec11299

[39] Lundgren JG, Gassmann AJ, Bernal J, Duan JJ, Ruberson J (2009) Ecological compatibility of GM crops and biological control. Crop Prot 28: 1017–1030.

[40] Tang JD, Collins HL, Metz TD, Earle ED, Zhao JZ, et al (2001) Greenhouse tests on resistance management of Bt transgenic plants using refuge strategies. J Econ Entomol 94: 240–247. 11233120 10.1603/0022-0493-94.1.240

[41] Roush RT (1998) Two toxin strategies for management of insecticidal transgenic crops: can pyramiding succeed where pesticide mixtures have not? Phil Trans Royal Soc Lond B 353: 1777–86.

[42] Shelton AM, Tang JD, Roush RT, Metz TD, Earle ED (2000) Field tests on managing resistance to Bt-engineered plants. Nat Biotechnol 18: 339–342. 10700153 10.1038/73804

[43] Zhao JZ, Cao J, Collins HL, Bates SL, Roush RT, et al (2005) Concurrent use of transgenic plants expressing a single and two *Bacillus thuringiensis* genes speeds insect adaptation to pyramided plants. Proc Natl Acad Sci USA 102: 8426–8430. 15939892 10.1073/pnas.0409324102 PMC1150809

[44] Metz TD, Roush RT, Tang JD, Shelton AM, Earle ED (1995) Transgenic broccoli expressing a *Bacillus thuringiensis* insecticidal crystal protein: implications for pest resistance management strategies. Mol Breeding 1: 309–317.

[45] Zhao J-Z, Collins HL, Shelton AM (2010) Testing insecticide resistance management strategies: mosaic versus rotations. Pest Manag Sci 66: 1101–1105. 20552665 10.1002/ps.1985

[46] SPSS (1998) SPSS User's Guide. Chicago, SPSS Inc.

